# Prey interception drives web invasion and spider size determines successful web takeover in nocturnal orb-web spiders

**DOI:** 10.1242/bio.012799

**Published:** 2015-09-24

**Authors:** Wenjin Gan, Shengjie Liu, Xiaodong Yang, Daiqin Li, Chaoliang Lei

**Affiliations:** 1 Hubei Insect Resources Utilization and Sustainable Pest Management Key Laboratory, Huazhong Agricultural University, Wuhan, Hubei 430070, China; 2 Key Laboratory of Tropical Forest Ecology, Xishuangbanna Tropical Botanical Garden, Chinese Academy of Sciences, Menglun, Mengla, Yunnan 666303, China; 3 Key Laboratory of Vegetation Restoration and Management of Degraded Ecosystems, South China Botanical Garden, Chinese Academy of Sciences, Guangzhou 510650, China; 4 Department of Biological Sciences, National University of Singapore, 14 Science Drive 4, 1117543, Singapore

**Keywords:** Conspecific competition, Web invasion, Intruder, Nocturnal, Prey abundance, Orb spiders

## Abstract

A striking feature of web-building spiders is the use of silk to make webs, mainly for prey capture. However, building a web is energetically expensive and increases the risk of predation. To reduce such costs and still have access to abundant prey, some web-building spiders have evolved web invasion behaviour. In general, no consistent patterns of web invasion have emerged and the factors determining web invasion remain largely unexplored. Here we report web invasion among conspecifics in seven nocturnal species of orb-web spiders, and examined the factors determining the probability of webs that could be invaded and taken over by conspecifics. About 36% of webs were invaded by conspecifics, and 25% of invaded webs were taken over by the invaders. A web that was built higher and intercepted more prey was more likely to be invaded. Once a web was invaded, the smaller the size of the resident spider, the more likely its web would be taken over by the invader. This study suggests that web invasion, as a possible way of reducing costs, may be widespread in nocturnal orb-web spiders.

## INTRODUCTION

Competition is an interaction between organisms or species, in which the fitness of one is lowered by the presence of another. Competition occurs in a wide range of generalist predators and is perceived by many researchers to be common among spiders (Lee and Klasing, 2004; Wise, 2006). The striking feature of web-building spiders is the use of silk to make webs mainly for prey capture (Foelix, 2011). Competition for web-building spiders not only involves competition over prey, but also over suitable websites and possibly the web itself (Eichenberger et al., 2009). Web-building spiders are known to invade the webs of conspecifics and displace them from the web (Wise, 2006). Web invasion is linked to competition for a web itself and for space among adult spiders (Hoffmaster, 1986). Some species of web-building spiders are expected to take over a web of other spiders rather than build its own web, because building a web is not only energetically expensive and time-consuming, but also greatly increases predation risk (Wise, 1983). For example, when spiders were released onto webs of heterospecifics, *Linyphia triangularis* (Araneae: Linyphiidae) was more likely to take over or share webs of *Frontinella communis* than the reverse (Houser et al., 2014). On the other hand, an existing web is a sign of a potentially good site and is an already constructed foraging device (Harwood et al., 2003). Therefore, it is often assumed that if the web value is determined by prey intake, web invasion would be more often present at prey-rich sites (Harwood et al., 2003; Glover, 2013; Houser et al., 2014).

Research on web invasion in web-building spiders has produced a wide range of results (Eichenberger et al., 2009). A few studies have shown that web invasion rarely occurs in web spiders (Enders, 1974; Wise, 1983), but other biologists propose that web invasion might exist in a wide range of web-building spiders (Christenson, 1984; Riechert and Gillespie, 1986). Recently, the results from laboratory experiments showed that the alien sheet-web spider *Mermessus trilobatus* was introduced from North America to Central Europe and has become locally abundant within the past three decades (Schmidt et al., 2008). The invading *M. trilobatus* is superior to smaller-sized immature native spiders in its ability to take over webs, thereby threatening populations of native spiders (Eichenberger et al., 2009). In general, no consistent patterns of web invasion have emerged and the factors determining web invasion remain largely unexplored. In this study, we used nocturnal orb-web spiders as a model system to address two specific questions: (i) is the web-invading behaviour widespread in nocturnal orb-weaving spiders, and (ii) if so, what are the factors determining web invasion and the success of web takeover?

## RESULTS

Our results showed that 36% (28 out of 77) of webs studied were invaded by the conspecifics, and 25% (7 out of 28) of the invaded webs were successfully taken over by the invaders among seven species of orb-web spiders (supplementary material Table S1; Figs S1a, S2a). Although the frequencies of web invasion and web takeover vary greatly among seven species, no statistically significant differences were observed among them (Table 1; supplementary material Figs S1, S2).

**Table 1.**
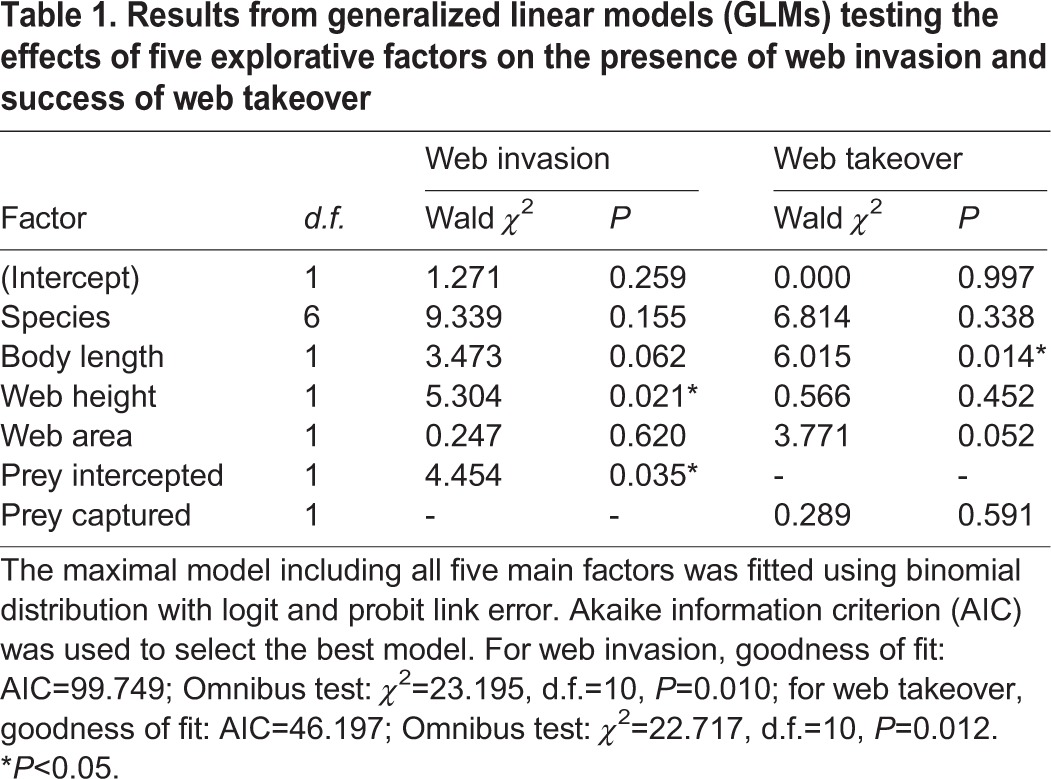
**Results from generalized linear models (GLMs) testing the effects of five explorative factors on the presence of web invasion and success of web takeover**

**Fig. 1.**
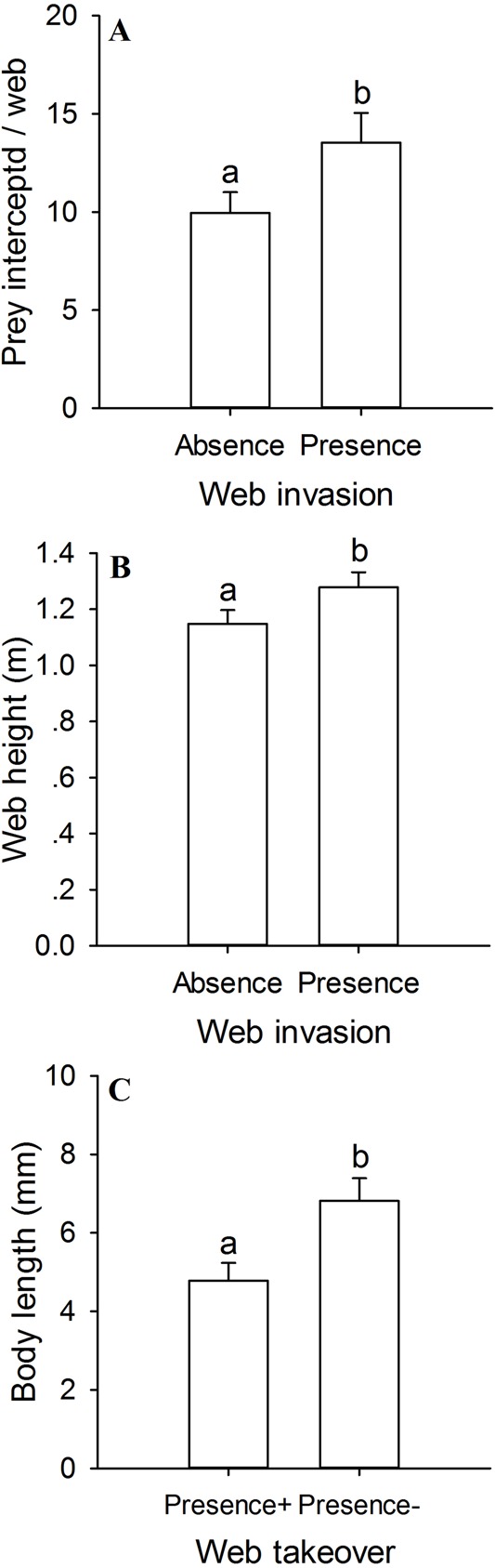
**Factors affecting web invasion and web takeover.** (A) Number of prey intercepted; and (B) web height of the invaded (Presence; *n*=28) and non-invaded webs (Absence; *n*=49), as well as (C) body size of the spiders whose webs were invaded and taken over (Presence+; *n*=7) and the spiders whose webs were invaded but not taken over (Presence−; *n*=21). Data are expressed as mean±s.e.m.; different lower-case letters indicate significant difference (*P*<0.05).

Results from generalised linear models (GLMs) revealed a significant main effect of five explorative factors on the probability of web invasion and web takeover (Table 1). The number of prey intercepted and web height were better predictors of the probability of a web being invaded by a conspecific: a web built higher that intercepted more prey was more likely to be invaded (Table 1; Fig. 1A,B). Spider species, body size and web size were poor predictors of the probability of web invasion (Table 1; supplementary material Fig. S3). Furthermore, spider size was the only predictor of the probability of an invaded web that could be taken over by the invader: the smaller the resident spider was, the more likely its web could be taken over by the invader (Table 1; Fig. 1C; supplementary material Fig. S4).

## DISCUSSION

This study suggests that web invasion may be common in nocturnal orb-web spiders. We also provide evidence that the abundance of available prey and web height were the better predictors for a spider invading a conspecific's web, and once it has invaded the web, the size of the resident spider will then determine the success of web takeover.

The factor considered to be most influential to web spiders when selecting a habitat is prey availability (Glover, 2013). Many spiders are known to strongly respond to prey abundance by choosing sites with abundant prey (Harwood et al., 2003; Thevenard et al., 2004). Meanwhile, many orb-web spiders relocate their webs in response to low rates of prey arrival (Nakata et al., 2003; Nakata and Ushimaru, 2004; Miyashita, 2005). In the present study, the invaded webs had intercepted more prey than the uninvaded webs which indicates that the existing webs may be good foraging sites. Spiders are unlikely to determine prey availability prior to web invasion. It seems unlikely that the webs invaded by the spiders during this experiment are in direct response to prey abundance. Instead, the spiders might use microclimatic cues, which in turn may indicate prey abundance (Prokop and Gryglakova, 2005). Another possible explanation for invaded webs having intercepted more prey is that these tested spiders may be able to respond to prey interception of other webs. Spiders usually concentrate in prey-rich areas (Harwood et al., 2003), and in our study site, they can attain high abundances in small patches (distance among webs about 2 cm, W.G., personal observation). Web-building spiders are sensitive to vibratory stimulation, thus spiders could sense the vibratory cues that indicate the value of neighbouring webs in this highly abundant area (Thevenard et al., 2004).

Our results showed that web height is important in determining web invasion in nocturnal orb-web spiders. Web design, such as mesh height, capture thread length and web area was affected by web height (Prokop and Gryglakova, 2005). Web area, capture thread length and mesh height were significantly related to number of prey intercepted (Blackledge et al., 2011). Therefore, web height could indirectly affect a spider's foraging success, whereby different heights have different microclimatic conditions, especially wind and light (Herberstein and Fleisch, 2003). These microclimatic changes affect insect mobility, indirectly influencing prey capture rate of a spider's web (Prokop and Gryglakova, 2005). A higher built web is a sign of a potentially good site and is more likely to be invaded. Another possible explanation is that moths are a dominant source of prey for nocturnal spiders (Prokop, 2006), and it is probable that a higher web would more accurately match the flying height of moths. Thus a higher web would intercept more prey and be more likely to be invaded.

Resident spider size is of great significance in affecting the success of web takeover. The smaller the resident spider, the more likely its web would be taken over by the invader. While size is a well-known correlate of competitive advantage in spiders, both between conspecifics and heterospecifics (Bednarski et al., 2010; Heiling and Herberstein, 1999; Houser et al., 2014). For example, large body size is associated with fighting success in *Misumenoides formosipes* (Dodson and Beck, 1993). In addition to large size, competitive ability, and the aggressive nature of invasive spiders (*Linyphia triangularis*) often allows them to take over webs of native spiders (*Frontinella communis*). Competition between invasive spiders and native spiders for both webs and web sites may contribute to the decline of native spiders (Bednarski et al., 2010; Houser et al., 2014). In the most extreme instances, web takeovers also result in the usurper preying upon the host (Eichenberger et al., 2009; Heiling and Herberstein, 1999). In this study, smaller spiders may abandon their webs to reduce the detrimental costs of interference competition.

In conclusion, our field study reveals that web invasion is widespread in nocturnal orb-web spiders. Prey availability and web height are important in affecting web invasion, and web resident spider size is crucial in taking over the web once the web was invaded. Web invasion in nocturnal orb-web spiders can influence the community structure of spiders. In consequence, understanding the web invasion behaviour is critical to predicting the population dynamic of nocturnal orb-web spiders in ecosystems.

## MATERIALS AND METHODS

### Study site and subjects

The study was carried out in a tropic rainforest near Menglun Village, Xishuangbanna Tropic Botanical Garden, Yunnan Province, China. All field observations were conducted from June to August in 2012. Seven species of nocturnal orb-web spiders (*n*=77; supplementary material Table S1, Fig. S1) were used: *Araneus dehaani* (*n*=10), *Araneus inustus* (*n*=13), *Araniella displicata* (*n*=13), *Lariniaria argiopiformis* (*n*=10), *Neoscona punctigera* (*n*=11), *Tetragnatha maxillosa* (*n*=10), and *Zygiella x-notata* (*n*=10). We followed the Association for the Study of Animal Behaviour/Animal Behavior Society Guidelines for the Use of Animals in Research (2006) published on the Animal Behaviour website, the legal requirements in China where the work was carried out.

### Experimental procedure

After the functional (i.e. prey-capture) web was completely finished we measured the body length of the spider, web height (the height of web location) from its hub to the ground (Bush et al., 2008), and web traits for estimating web capture area as described in other studies (Herberstein and Tso, 2000; Blackledge et al., 2011). We used infrared video cameras (Sony HDR-XR550E) to record simultaneously four individual webs of spiders each night. We recorded web invasion and takeover events between 20:00 h and 07:30 h. To minimize possible interference, video cameras were placed 1–2 m away from the spiders and their webs. Only females were used, and each spider was used only once. We marked the sites where spiders built their webs, and we changed filming location every day. When the field experiments were completed we viewed the video footage in the laboratory at Xishuangbanna Tropical Botanical Garden, Yunnan, China. The video footage repeated playback four times, and we noted the follow events for each spider: (i) the occurrence of web invasion; (ii) if so, the success of web takeover; and (iii) the number of prey intercepted and prey captured by the web. In this field observation, a total of 651 h of video recordings was made and used in data analysis.

### Data analyses

We analysed the data using generalised linear models (GLMs). When analysing the data on the frequency of web invasion, we used web invasion (presence/absence) as the dependent variable, and five explorative factors (species, body length, web height, web area, number of prey intercepted) as predictors. When analysing the data on the frequency of web takeover, we used web takeover (success/failure) as the dependent variable, and five explorative factors (species, body length, web height, web area, number of prey captured) as predictors. For both GLM analyses, the maximal models including all five factors were fitted using binomial distribution with logit and probit link error. Akaike information criterion (AIC) was used to select the best model. All statistical analyses were performed using IBM SPSS 22.0.
